# Management of Severe Behçet’s Disease

**DOI:** 10.1007/s11926-026-01217-z

**Published:** 2026-03-31

**Authors:** Kerem Abacar, Haner Direskeneli, Fatma Alibaz-Oner

**Affiliations:** 1 Leeds Institute of Rheumatic and Musculoskeletal Medicine, University of Leeds, Leeds, UK; 2 School of Medicine, Department of Internal Medicine, Division of Rheumatology, Marmara University, Istanbul, Türkiye

## Abstract

**Purpose of Review:**

Behçet’s disease (BD) is a multisystemic inflammatory disorder in which treatment decisions are largely driven by the pattern and severity of organ involvement. This review provides a practical, organ-based overview of current therapeutic strategies for severe and refractory BD, with an emphasis on treatment selection and timing in routine clinical practice.

**Recent Findings:**

Recent evidence supports an upfront, intensive treatment approach for life-threatening or damage-prone organ involvement, particularly neurological, vascular, and ocular disease, where delays in inflammation control are closely associated with irreversible damage. Anti–tumour necrosis factor agents form the core of therapy in these settings, supported by the strongest disease-specific data, while other biologic and targeted therapies are increasingly used in refractory cases. In contrast, mucocutaneous and articular manifestations predominantly affect quality of life and are commonly managed with stepwise strategies. Colchicine remains a widely used first-line treatment in routine practice and provides effective control of mucocutaneous and joint manifestations in a substantial proportion of patients, with escalation to additional systemic or targeted therapies guided by persistence, refractoriness, and patient burden. Importantly, treatment responses vary across organ systems, and benefit in one domain cannot be assumed to translate to others, reinforcing the need for organ-specific treatment planning.

**Summary:**

Optimal management of BD depends on matching treatment intensity to organ-specific risk, prioritising early aggressive therapy for major organ involvement while adopting proportionate, stepwise approaches for non–life-threatening disease. Improved outcomes are likely to be achieved through timely recognition of patients at risk of progression and early use of appropriately targeted therapies to prevent irreversible organ damage.

## Introduction

Behçet’s disease (BD) is a chronic, relapsing, multisystemic inflammatory disease characterised by a wide spectrum of clinical manifestations involving the mucosa, skin, joints, eyes, nervous system, vascular structures, and gastrointestinal tract [1, 2]. Beyond the involvement of multiple organ systems, the disease is notable for the diversity of manifestations that may arise within each affected organ. The same organ system can present with clinically distinct phenotypes that differ substantially in severity, prognosis, and therapeutic implications [1–4]. Moreover, different organ manifestations frequently coexist or evolve sequentially within the same patient, contributing to the marked heterogeneity of disease trajectories observed in BD [1, 3].

The overall burden of BD extends beyond organ-specific morbidity and includes substantial impairment in health-related quality of life. Persistent disease activity, recurrent flares, and the unpredictable course of the disease negatively affect physical functioning, psychological well-being, and social participation [5, 6]. At the same time, long-term outcome is strongly influenced by the development of irreversible organ damage. Ocular, neurological, major vascular, and gastrointestinal involvement are consistently associated with permanent functional loss, disability, and increased mortality risk, and therefore represent the principal determinants of prognosis in BD [3, 4, 7]. This dual impact on quality of life and organ survival highlights the need to consider disease severity in more than a single clinical perspective.

Several disease activity and severity indices have been proposed to quantify disease burden in BD, including the Behcet Disease Activity Index, the Behcet Disease Current Activity Form, the Iranian Behcet Disease Dynamic Activity Measure, and cumulative severity scores such as the Krause total clinical severity score [8–11]. In addition, the Behçet’s Syndrome Overall Damage Index (BODI) has been developed to quantify irreversible organ damage accrual over time, providing a complementary framework that captures long-term disease consequences rather than inflammatory activity alone [12]. These composite instruments have been valuable for research and longitudinal assessment, however, their relevance to individual treatment decisions is less clear, particularly in patients whose overall disease burden is driven by a single dominant organ manifestation. As organ involvement in BD often occurs in a focal or compartmentalised manner, it remains uncertain to what extent such composite scores fully capture clinically relevant organ-specific risk in routine practice. This has contributed to ongoing uncertainty regarding their role in guiding individual treatment decisions, particularly in patients with heterogeneous or evolving patterns of organ involvement.

Although BD is thought to share a common pathogenic backbone, supported by strong associations with MHC class I–related pathways and a close interplay between cytotoxic CD8⁺ T cells and innate immune mechanisms [13–17], accumulating evidence suggests that the biological context of inflammation differs across affected organs. Tissue architecture, resident immune and stromal cell populations, and organ-specific physiological immune functions are likely to shape local inflammatory microenvironments, influencing dominant cytokine pathways, patterns of tissue injury, and response to therapy. These differences may help explain why efficacy and side-effect profiles of different therapeutic agents vary between disease domains, even within the same patient. In light of this clinical and biological heterogeneity, an organ-oriented approach to disease assessment and management is widely adopted in practice. Accordingly, in this review, we examine the management of severe BD on an organ-by-organ basis, with a particular focus on severe, refractory, and life-threatening manifestations.

## Mucocutaneus involvement

Mucocutaneous involvement is the most common clinical domain in BD and typically comprises recurrent oral aphthous ulcers, genital ulcers, erythema nodosum-like lesions, and papulopustular or acneiform eruptions [1, 3, 4]. These manifestations are rarely life-threatening and usually do not drive mortality risk. Nevertheless, they can be clinically severe in a different sense: frequent, painful, and persistent lesions often translate into a sustained disease burden with major impairment in daily functioning, psychological well-being, and social participation [3, 4, 6]. Patient-reported outcomes and quality-of-life studies consistently show that fluctuations in mucosal disease activity parallel meaningful changes in oral health–related and overall quality of life, supporting the idea that “severity” in this domain is primarily defined by refractoriness and cumulative impact rather than organ failure risk [4–6]. This distinction matters when framing management goals, because the therapeutic threshold is often determined by long-term burden and patient-centred outcomes, while still maintaining a proportionate approach to immunosuppression.

In line with this risk profile, guideline-based management of mucocutaneous disease generally favours a stepwise strategy, starting with topical measures and escalating according to persistence and disability [3, 18]. The EULAR recommendations support topical glucocorticoids (GC) for oral and genital ulcers and commonly use colchicine as a systemic first-line option, particularly when lesions are frequent or accompanied by erythema nodosum–like features [3, 4]. Short courses of systemic GCs are typically reserved for acute, highly symptomatic flares rather than as maintenance therapy [3]. For patients who remain symptomatic despite conventional steps, escalation increasingly favours targeted or biologic therapies when quality-of-life impairment is sustained and clearly attributable to refractory mucosal disease, rather than conventional immunosuppressive agents. Apremilast has the strongest randomised trial evidence among targeted agents for oral ulcers in BD and improves both ulcer outcomes and patient-reported measures [19]. Anti-TNF therapy is also used in refractory mucocutaneous disease, typically informed by broader BD management experience and the frequent coexistence of other inflammatory domains [3]. Thalidomide has demonstrated efficacy in severe refractory oral and genital ulceration in controlled studies and remains acknowledged in guideline frameworks [20]; however, the expanding evidence base for newer targeted therapies, together with thalidomide’s well-recognised cumulative neurotoxicity and teratogenicity [21], has led to its positioning as a later-line option reserved for carefully selected refractory cases. Beyond these, IL-17 inhibition and IL-12/23 pathway targeting have been explored mainly in refractory mucosal and articular phenotypes, with observational data suggesting potential benefit in selected patients, notably, dose escalation strategies have been reported for secukinumab, implying that this may be a domain where careful titration is sometimes applied in practice, although the evidence base remains less robust than for apremilast or anti-TNF approaches [22–27]. Overall, mucocutaneous BD is a setting in which a conservative escalation framework is rational, but where modern targeted options may be appropriate when refractory disease produces a sustained and clinically meaningful burden with treatment decisions primarily guided by patient-centred outcomes rather than organ-threatening risk (Table 1). In addition to pharmacological therapy, supportive lifestyle measures are routinely recommended for both oral and genital ulcers, including meticulous local hygiene, avoidance of mechanical trauma, optimisation of oral care (regular tooth brushing and dental follow-up), and individualised dietary modification to reduce known mucosal triggers, approaches that are consistently endorsed in expert guidance despite limited high-level trial evidence [3, 4, 28–30].

**Table 1:** Organ-specific considerations in the management of BD

Organ domain	First-line treatment	Primary severity drivers	Preferred treatment in severe / refractory disease	Key considerations
Mucocutaneous	Colchicine, topical GCs (oral/genital)	Persistent symptom burden, quality-of-life impairment	Apremilast, azathioprine, infliximab or adalimumab, IL-23 pathway–targeted therapies^*^	Stepwise escalation preferred, treatment decisions driven by cumulative disease burden rather than organ-threatening risk
Articular	Colchicine, NSAIDs, intra-articular glucocorticoids	Inflammatory activity, functional limitation	Methotrexate, sulfasalazine, infliximab or adalimumab	Non-erosive course common, local therapies often sufficient
Ocular	High-dose systemic glucocorticoids, azathioprine, infliximab, adalimumab	Posterior segment involvement, ischemic or vasculitic features	Adalimumab, infliximab, tocilizumab, Interferon alfa-2a^*^	Early escalation critical, speed and depth of suppression determine visual outcome
Neurological	High-dose systemic glucocorticoids, azathioprine, cyclophosphamide	Parenchymal involvement, extent of neurological deficits	Infliximab, cyclophosphamide, Interferon alfa-2a,	Early escalation often required in relapsing disease, anticoagulation reserved for selected CVST
Vascular	High-dose systemic glucocorticoids, cyclophosphamide	Major vessel involvement, pulmonary arterial disease	Infliximab, adalimumab, Interferon alfa-2a, JAK inhibitors ^*^	Vasculitis-first approach, endovascular or surgical intervention may be required in pulmonary artery involvement, anticoagulation individualised
Gastrointestinal	Azathioprine, systemic glucocorticoids	Deep ulcers, bleeding or perforation risk	Infliximab, adalimumab, JAK inhibitors	Management differs from classical inflammatory bowel disease; treatment response is heterogeneous

* IL-1 blockade (e.g. anakinra or canakinumab) has been described in highly selected refractory cases, supported by limited observational evidence

### (This table is original and was created by the authors for this review.)

#### Joint involvement

Articular involvement in BD is common, but it is typically non-erosive and non-deforming, presenting as recurrent, self-limited monoarthritis or oligoarthritis that most often affects large peripheral joints such as the knees and ankles [1, 31]. Prospective observations also support an intermittent pattern, with attacks that cluster in a small number of joints and generally resolve without lasting structural consequences [32]. For this reason, joint involvement is not usually regarded as a driver of life-threatening disease, and it is generally thought to lead to persistent functional impairment or long-term restriction of movement only rarely [1, 31]. Management is therefore usually pragmatic and stepwise: colchicine and non-steroidal anti-inflammatory drugs are commonly used first-line, with short courses of low-dose systemic GCs or intra-articular injections reserved for acute, highly symptomatic flares [3]. Escalation to conventional immunosuppressive agents such as methotrexate or sulfasalazine, or to biological therapies including infliximab or adalimumab, is uncommon for isolated peripheral arthritis and is more often considered in the context of multisystem disease or concomitant major organ involvement [3].

## Ocular Involvement

Ocular involvement in BD is usually discussed under the umbrella of “uveitis”, but in practice it is a package of inflammatory problems that can threaten vision through several mechanisms at once: recurrent non-granulomatous anterior uveitis (sometimes with hypopyon), intermediate uveitis (vitritis), posterior uveitis and panuveitis, and, most characteristically, occlusive retinal vasculitis with ischemia and secondary complications such as macular edema, retinal neovascularisation, and optic nerve involvement [1, 33, 34]. Ocular involvement is regarded as a major organ domain because uveitis is inherently relapsing, and repeated inflammatory episodes drive cumulative structural damage when inflammation is not durably controlled. Before earlier aggressive immunosuppression and the biologic era, historical cohorts and older series repeatedly reported substantial rates of severe visual loss and blindness over follow-up, often driven by posterior segment disease (retinal vasculitis, macular involvement) rather than anterior inflammation alone [35–37]. Even in more contemporary series, the burden has not disappeared, with recent reviews consistently reporting double-digit rates of severe visual loss or blindness, generally ranging from approximately 10% to 25% across cohorts, and emphasising that prognosis is closely linked to early control of posterior segment inflammation and relapse prevention [34–36].

For this reason, biologic therapy is now considered central to the management of severe ocular BD, as this is one of the few domains in which the speed and depth of inflammatory suppression directly determine long-term visual outcome. Monoclonal Anti-TNF therapy has the most extensive disease-specific evidence base. Infliximab was reported early as rapidly effective in refractory uveitis, followed by prospective and multicentre datasets showing marked reductions in attack frequency and steroid sparing, with visual outcomes generally stabilising when relapses are suppressed [38–44]. Adalimumab has also strong evidence in BD related uveitis, supported by real world and multicentre cohort studies demonstrating reduced flare rates, control of retinal vasculitis, and GC sparing effects [45–49] and this is also reinforced by randomised controlled data from the VISUAL I [50] and VISUAL II [51] trials, in which patients with BD were included in small numbers but showed consistent benefit in preventing disease flares and treatment failure. Interferon alfa-2a (IFNa) is another cornerstone option for sight-threatening posterior uveitis or retinal vasculitis, with classic cohorts showing high response rates and durable remissions in a subset, although its toxicity profile and logistics differ from anti-TNF strategies [37, 52, 53]. However, problems with drug availability limits its current use. Data on Pegylated IFNa is promising, however clinical data is limited.

Beyond these, cytokine-targeted therapies have been explored in refractory disease. IL-1 inhibition with agents such as anakinra and canakinumab has been reported in observational cohorts and case series, with some signals of benefit in selected patients with refractory ocular and systemic inflammation. However, the phase III randomised trial of gevokizumab in Behçet’s uveitis did not meet its primary efficacy endpoint, casting doubts on the role of IL-1 blockade in ocular BD. Accordingly, IL-1 inhibition cannot be considered standard therapy and, if used, should be restricted to highly selected refractory cases after failure of established treatments [54–57]. IL-6 receptor blockade with tocilizumab has accumulated multicentre evidence particularly for uveitic macular edema and refractory ocular disease, including comparative data versus anti-TNF in this complication-heavy subgroup [58–60]. Finally, “second-line” anti-TNF options such as golimumab and certolizumab pegol are supported by observational cohorts and rescue-therapy series, mainly in patients who failed or could not tolerate first anti-TNF monoclonal agents [61–64]. In severe ocular Behçet’s disease, severity most often reflects posterior involvement with a relapsing and damage-prone course. In this setting, prompt initiation of high-dose GCs in combination with biologic therapy is essential, and biologics should be regarded as disease-modifying treatment rather than a rescue option (Table 1).

## Neurological Involvement

Neurological involvement in BD is uncommon but strongly associated with long-term disability. In the largest dedicated clinical series, Akman-Demir et al. analysed 200 patients with neuro-BD, parenchymal central nervous system involvement was present in 162 patients (81%), while non-parenchymal disease was observed in 38 patients (19%) [1]. Within the parenchymal group, brainstem or brainstem-plus involvement was the predominant pattern, reported in 83 of 162 patients (51.2%), followed by hemispheric involvement in 25 patients (15.4%) and spinal cord disease in 23 patients (14.2%) [65, 66]. Hemiparesis occurred in 82 patients, cranial nerve involvement in 71, dysarthria in 58, and behavioural or cognitive disturbance in 43 patients, illustrating that focal neurological deficits rather than isolated headache syndromes dominate the clinical picture [65, 66].

Disease course is frequently relapsing, and damage driven. In a French multicentre cohort of 115 patients with parenchymal neuro BD, all patients received high dose GCs for acute attacks, cyclophosphamide was used in 53 patients (46.1%), while azathioprine was prescribed in 40 patients (34.8%) as maintenance therapy, suggesting that azathioprine is sufficient for long term disease control in a substantial proportion of patients, whereas cyclophosphamide appears more effective during the early phase of severe disease, with longer term outcomes converging over time [67]. During a median follow-up of 73 months, 29 patients (25.2%) developed severe dependency or died, underlining the cumulative burden of neurological damage [67]. More recent randomised controlled evidence has further informed treatment strategies for severe disease. In a phase 2 multicentre trial by Saadoun et al., infliximab was compared with cyclophosphamide in 52 patients with severe BD, including 15 patients with neuro BD involvement. At week 22, complete response was achieved in 81% of patients treated with infliximab compared with 56% of those receiving cyclophosphamide, supporting infliximab as an effective early induction option in severe or relapsing parenchymal neurological disease [68].

Non-parenchymal neurological involvement is dominated by cerebral venous sinus thrombosis, which accounted for 38 of 200 neuro-BD cases in a study [65]. In this setting, immunosuppression with GCs is central, and current EULAR recommendations support the use of anticoagulation in CVST when bleeding risk is low and pulmonary artery aneurysms have been excluded, reflecting accumulated cohort experience rather than randomized data [3]. Overall, management of neuro-BD increasingly favours early escalation to biologic therapy, most notably infliximab, for severe or relapsing parenchymal involvement, alongside sustained immunosuppression for relapse prevention, while anticoagulation in cerebral venous sinus thrombosis remains individualised and adjunctive to inflammation control (Table 1).

## Intestinal Involvement

Intestinal involvement in BD refers to objective gastrointestinal ulceration attributable to the disease, most characteristically deep, round or oval ulcers with discrete margins and a strong predilection for the ileocecal region [69, 70]. Clinical presentation ranges from chronic abdominal pain and diarrhoea to overt gastrointestinal bleeding, and in a subset of patients progresses to severe complications such as perforation, fistulisation, obstruction, or the need for urgent surgery [70–72]. Older cohorts and longitudinal studies already highlighted intestinal BD as a prognostically relevant phenotype, characterised by a relapsing course and a substantial risk of cumulative bowel damage [71–74]. Even when initial inflammatory episodes respond to treatment, relapse is common, and postoperative recurrence rates reported in dedicated series underscore that insufficient disease control may translate into repeated surgical interventions rather than isolated symptomatic flares [73–75].

From a therapeutic perspective, mild intestinal disease may be managed with 5-aminosalicylates and/or short courses of systemic glucocorticoids for symptom control, with azathioprine commonly considered in patients with relapsing or steroid-dependent disease, in line with current EULAR recommendations [3], but contemporary guidance emphasises early escalation in patients with moderate to severe activity, high-risk ulcer morphology, steroid dependence, or refractory disease [71, 72]. Among biologics, anti–tumour necrosis factor therapy has the strongest and most disease-specific evidence base in intestinal BD. Infliximab has been evaluated in prospective studies and large multicentre cohorts, demonstrating meaningful clinical and endoscopic improvement in patients with active intestinal disease refractory to conventional therapy, with response rates generally reported in the range of approximately 60–80% during induction and early maintenance phases [76–78]. Adalimumab has similarly been supported by phase 3 trial data and long-term observational follow-up, including a large real-world cohort of 462 patients, in which around 70% of patients achieved sustained clinical response or remission, alongside an acceptable long-term safety profile [79, 80]. Beyond first-line anti-TNF strategies, the evidence becomes more heterogeneous and largely observational. Thalidomide has been repeatedly reported as an option in selected refractory cases, while newer targeted therapies, including vedolizumab have been explored mainly as rescue treatments in highly pre-treated patients [81, 82]. Small retrospective case series and pilot cohorts, predominantly involving tofacitinib and baricitinib, have reported meaningful clinical and, in some cases, endoscopic improvement in patients with refractory intestinal BD after failure of conventional and anti-TNF therapy [83–86]. Importantly, data on JAK inhibitors in intestinal BD are conflicting, with some reports suggesting benefit and others indicating limited efficacy or lack of sustained response [87], this variability highlights both the heterogeneity of intestinal disease mechanisms and the current uncertainty surrounding optimal positioning of these agents. Overall, intestinal BD is a setting in which the clinical value of biologic therapy extends beyond symptom relief, as sustained inflammatory control may directly reduce the risk of cumulative bowel damage and the need for repeated surgical intervention (Table 1).

### Vascular Involvement

Vascular involvement is one of the clearest places where “severe” BD is not a descriptive qualifier but a marker of prognosis. The vascular spectrum spans inflammatory venous thrombosis (most commonly lower extremity deep vein thrombosis), major vein disease (inferior or superior vena cava thrombosis, hepatic vein thrombosis leading to Budd–Chiari syndrome, intracardiac thrombosis), and arterial disease (pulmonary artery involvement with thrombosis and or aneurysms, aortic or peripheral arterial aneurysms) [88]. These phenotypes differ not only in anatomy but also in clinical course and lethality. Pulmonary artery aneurysms, in particular, have long been recognised as a major driver of mortality, typically through massive haemoptysis, and classic series and reviews emphasise the need for urgent immunosuppression rather than a “standard VTE” mindset [89, 90]. In venous disease, the dominant clinical problem is often relapse and chronic morbidity. Even when limb-threatening consequences are less common than in non-BD thrombosis cohorts, post-thrombotic syndrome and venous disease specific quality of life impairment remain clinically meaningful, and correlate with overall disease activity, supporting the idea that controlling vascular inflammation is central to long term outcome [91]. Large cohort studies also show that relapse of lower extremity deep vein thrombosis is frequent despite conventional immunosuppression, whereas more targeted immunosuppressive therapies appear more effective in preventing recurrence in selected patients, reinforcing that vascular BD reflects vasculitic inflammation rather than hypercoagulability alone [92].

Therapeutically, modern guidance is consistently organ oriented: for severe vascular phenotypes, induction with high dose glucocorticoids plus an immunosuppressive agent is prioritised, with escalation to biologic therapy when rapid control is needed, when there is major vessel disease, or when disease is refractory [3, 4]. Historically, cyclophosphamide combined with high dose glucocorticoids has been the default induction strategy for life threatening vascular BD, particularly pulmonary artery aneurysm and other rapidly fatal phenotypes, based largely on older retrospective series in which pulsed or oral cyclophosphamide plus GCs was the dominant approach. This practice pattern is also reflected in contemporary guideline frameworks that prioritise glucocorticoids with an immunosuppressive agent for major vessel involvement, reserving biologic escalation for severe or refractory presentations [90].

The strongest randomised evidence to date supporting a biologic-first approach in severe disease comes from the head-to-head trial comparing infliximab with cyclophosphamide for severe BD with vascular and or central nervous system involvement. In that trial, infliximab achieved a higher complete response rate at week 22 and was associated with fewer adverse events, directly challenging the historical assumption that cyclophosphamide is the default induction strategy for life threatening BD [68]. This RCT sits alongside a substantial body of observational data showing high remission rates of anti-TNF therapy in severe and refractory major vessel disease, including series focused on major vessel involvement and broader anti-TNF cohorts that include vascular phenotypes [93, 94]. For Budd–Chiari syndrome related to BD, outcomes have historically been poor in older series, and contemporary practice increasingly treats it as a true emergency vasculitis phenotype, combining aggressive immunosuppression with early multidisciplinary input. Small case series suggest that infliximab can induce remission in hepatic vein thrombosis, and larger outcome surveys frame Budd–Chiari as one of the most prognostically serious vascular presentations [95, 96]. The anticoagulation question remains clinically important but should be framed carefully: cohort data support immunosuppression as the key relapse-preventive therapy for venous thrombosis, while the incremental value of anticoagulation likely varies by phenotype and bleeding risk. In pulmonary arterial involvement, a recent large cohort suggested that adding anticoagulation to immunosuppressive therapy may reduce relapse, but this must be balanced against haemoptysis risk, particularly when aneurysmal disease is present, and most recommendations still place inflammation control at the centre of decision-making [97, 98]. In selected patients with pulmonary vascular involvement, chronic thromboembolic pulmonary hypertension has been managed with pulmonary endarterectomy in experienced centres, while life-threatening haemoptysis related to pulmonary artery aneurysms may require endovascular embolization (coils, glue or vascular plugs) as an adjunct to urgent immunosuppression [99–101]. In refractory vascular disease, emerging targeted options are being explored beyond anti TNF therapy, although the evidence base remains limited and organ specific. For IL 6 receptor blockade, a single centre observational cohort suggested that tocilizumab can be effective for refractory arterial lesions with steroid and immunosuppressant sparing [102], but other real world series have reported unfavourable vascular outcomes, underscoring the need for careful patient selection and close imaging based monitoring [103]. In parallel, JAK inhibition has early supportive data in highly pre-treated patients, including a pilot study of baricitinib in refractory vascular and cardiac BD reporting high short term complete response rates and a glucocorticoid sparing effect [104], but these findings still require validation in larger, comparative cohorts. In practice, vascular BD is best approached by recognising that prognosis is driven by organ-specific risk, while treatment decisions hinge on effective control of vascular inflammation, with antithrombotic strategies considered on an individual basis rather than applied uniformly (Table 1).

## General perspective on severity and early intervention

Beyond organ-specific management, an overarching challenge in BD lies in recognising disease trajectories early enough to prevent progression toward irreversible organ damage. Although the distinction between “severe” and “non-severe” disease is clinically useful, accumulating evidence suggests that severity in BD is better conceptualised as a dynamic spectrum rather than a fixed category. Large longitudinal cohort data demonstrate that relapses and new major organ involvement frequently occur despite conventional immunosuppressive therapy, and that these events are significantly less common under biologic treatment, supporting the concept that earlier and more intensive intervention may modify disease course rather than merely treat established severity [105]. From this perspective, waiting for “severe disease” to declare itself may represent a missed therapeutic window. Earlier risk stratification and timely escalation of therapy in patients with features associated with a more aggressive trajectory may therefore reduce progression toward organ-threatening involvement.

## Organ-specific heterogeneity and differential treatment responses

An additional complexity in the management of BD is that therapeutic efficacy is not uniform across organ systems, and no single agent can be assumed to be universally effective across all disease domains. Clinical experience and cohort data indicate that treatments highly effective for one organ may show limited or unfavourable effects in others. In a pilot series evaluating tofacitinib in refractory BD, vascular and articular manifestations improved overall, whereas gastrointestinal involvement frequently persisted or worsened, illustrating a dissociation between systemic inflammatory control and intestinal disease response [87]. Similarly, IL-6 receptor blockade has been associated with poor outcomes in gastrointestinal BD, raising concerns regarding its use in patients with active mucosal disease despite efficacy in other inflammatory settings [106]. Paradoxical BD-like manifestations and disease exacerbations have also been reported under IL-17 A inhibition, underscoring that targeted cytokine blockade may differentially modulate pathogenic pathways rather than uniformly suppress disease activity [107, 108]. Even within anti-TNF therapy, organ-specific divergence is observed, with large observational cohorts showing the emergence of new disease manifestations during infliximab treatment despite adequate control of the index organ [109]. These observations underscore the need for organ-aware therapeutic strategies and ongoing reassessment across disease domains. In clinical practice, inappropriate extrapolation of efficacy from one organ system to another may not only limit benefit, but in some settings actively worsen outcomes.

## Conclusion

Management of BD should be guided by an organ-driven treatment strategy that aligns therapeutic intensity with the risk of irreversible damage. Life-threatening or damage-prone involvement, particularly of the nervous system, major vessels, and the eye, requires early and intensive immunosuppression with prompt escalation to biologic therapy to achieve rapid and sustained disease control. In contrast, mucocutaneous and articular manifestations, which predominantly impair quality of life, are often effectively managed with stepwise approaches based on colchicine, topical measures, and low-dose systemic therapies, with escalation reserved for refractory or persistently burdensome disease. Given the unpredictable course of BD, close monitoring and timely adjustment of therapy are essential, and applying appropriately targeted treatments at the right time is likely to be key to preventing progression toward organ-threatening involvement and improving long-term outcomes. 

## Key References

- Hatemi G, Christensen R, Bang D, Bodaghi B, Celik AF, Fortune F, Gaudric J, Gul A, Kotter I, Leccese P, Mahr A, Moots R, Ozguler Y, Richter J, Saadoun D, Salvarani C, Scuderi F, Sfikakis PP, Siva A, Stanford M, Tugal-Tutkun I, West R, Yurdakul S, Olivieri I, Yazici H, 2018 update of the EULAR recommendations for the management of Behcet’s syndrome. Ann Rheum Dis.2018;77(6):808 − 18.

- ◦ The most widely cited organ-based guideline forming the backbone of contemporary management across all Behçet phenotypes, including severe major-organ disease.

- Ozguler Y, Leccese P, Christensen R, Esatoglu SN, Bang D, Bodaghi B, Çelik AF, Fortune F, Gaudric J, Gul A, Kötter I, Mahr A, Moots RJ, Richter J, Saadoun D, Salvarani C, Scuderi F, Sfikakis PP, Siva A, Stanford M, Tugal-Tutkun I, West R, Yurdakul S, Olivieri I, Yazici H, Hatemi G. Management of major organ involvement of Behçet's syndrome: a systematic review for update of the EULAR recommendations. Rheumatology (Oxford). 2018;57(12):2200–12.

-  ◦ A focused systematic review directly supporting therapeutic decision-making in severe Behçet’s disease with major organ involvement.

- International Team for the Revision of the International Criteria for Behcet's D. The International Criteria for Behcet's Disease (ICBD): a collaborative study of 27 countries on the sensitivity and specificity of the new criteria. J Eur Acad Dermatol Venereol. 2014;28(3):338-47.

- ◦ The most frequently used international classification criteria, ensuring standardized case definition across clinical studies and cohorts.

- Saadoun D, Maalouf G, Vieira M, Trad S, Lazaro E, Sacre K, Plessier A, Sene T, Kone-Paut I, Noel N, Mekinian A, Lambert M, Ribeiro E, Mirault T, Mele N, Dellal A, Fain O, Melki I, Chiche L, Gaudric J, Redheuil A, Maillart E, Ghembaza A, Desbois AC, Mirouse A, Domont F, Leroux G, Ferfar Y, Rigolet A, Viallard JF, Vautier M, Resche-Rigon M, Cacoub P. Infliximab versus Cyclophosphamide for Severe Behcet's Syndrome. NEJM Evid. 2024;3(11):EVIDoa2300354.

- ◦ A landmark head-to-head comparative study informing escalation strategies in severe vascular and neurological Behçet’s disease.

- Bettiol A, Alibaz-Oner F, Direskeneli H, Hatemi G, Saadoun D, Seyahi E, Prisco D, Emmi G.Vascular Behcet syndrome: from pathogenesis to treatment. Nat Rev Rheumatol. 2023;19(2):111-26.

- ◦ A high-impact state-of-the-art review linking clinical vascular phenotypes with evidence-based treatment strategies in high-morbidity disease.

- Hatemi G, Mahr A, Ishigatsubo Y, Song YW, Takeno M, Kim D, Melikoglu M, Cheng S, McCue S, Paris M, Chen M, Yazici Y.Trial of Apremilast for Oral Ulcers in Behcet's Syndrome. N Engl J Med. 2019;381(20):1918-28.

- ◦ A pivotal randomized controlled trial demonstrating the efficacy of targeted therapy in mucocutaneous Behçet’s disease.

- Tukek NB, Esatoglu SN, Hatemi G, Caliskan EB, Ozyazgan Y, Ucar D, Ozguler Y, Seyahi E, Melikoglu M, Uygunoglu U, Siva A, Kutlubay Z, Hatemi I, Celik AF, Ugurlu S, Fresko I, Yurdakul S, Yazici H, Hamuryudan V. Emergence of new manifestations during infliximab treatment in Behcet's syndrome. Rheumatology (Oxford).2022;61(9):3746-53.

- ◦ A real-world study highlighting paradoxical and newly emerging disease manifestations during anti-TNF therapy in Behçet’s syndrome.

- Bozkurt T, Karabacak M, Karatas H, Kutlug Agackiran S, Ergun T, Direskeneli H, Alibaz-Oner F.Earlier and more aggressive treatment with biologics may prevent relapses and further new organ involvement in Behcet's disease. Clin Immunol. 2023;248:109263.

- ◦ This multicenter observational study supports early and intensive biologic treatment to reduce relapse risk and prevent accumulation of new organ involvement.

- Kone-Paut I, Barete S, Bodaghi B, Deiva K, Desbois AC, Galeotti C, Gaudric J, Kaplanski G, Mahr A, Noel N, Piram M, Tran TA, Wechsler B, Saadoun D, Collaborators.French recommendations for the management of Behcet's disease. Orphanet J Rare Dis. 2021;16(Suppl 1):352.

- ◦ A comprehensive national consensus complementing EULAR recommendations with pragmatic management algorithms for refractory and complex cases.

## Data Availability

No datasets were generated or analysed during the current study.
